# Systematic generation of biophysically detailed models with generalization capability for non-spiking neurons

**DOI:** 10.1371/journal.pone.0268380

**Published:** 2022-05-13

**Authors:** Loïs Naudin, Juan Luis Jiménez Laredo, Qiang Liu, Nathalie Corson

**Affiliations:** 1 Department of Applied Mathematics, Normandie University, Le Havre, Normandie, France; 2 Department of Computer Sciences, Normandie University, Le Havre, Normandie, France; 3 Department of Neuroscience, City University of Hong Kong, Kowloon, Hong Kong, SAR, China; Key Laboratory for NeuroInformation of Ministry of Education, School of Life Science and Technology, University of Electronic Science and Technology of China, CHINA

## Abstract

Unlike spiking neurons which compress continuous inputs into digital signals for transmitting information via action potentials, non-spiking neurons modulate analog signals through graded potential responses. Such neurons have been found in a large variety of nervous tissues in both vertebrate and invertebrate species, and have been proven to play a central role in neuronal information processing. If general and vast efforts have been made for many years to model spiking neurons using conductance-based models (CBMs), very few methods have been developed for non-spiking neurons. When a CBM is built to characterize the neuron behavior, it should be endowed with generalization capabilities (*i.e*. the ability to predict acceptable neuronal responses to different novel stimuli not used during the model’s building). Yet, since CBMs contain a large number of parameters, they may typically suffer from a lack of such a capability. In this paper, we propose a new systematic approach based on multi-objective optimization which builds general non-spiking models with generalization capabilities. The proposed approach only requires macroscopic experimental data from which all the model parameters are simultaneously determined without compromise. Such an approach is applied on three non-spiking neurons of the nematode *Caenorhabditis elegans* (*C. elegans*), a well-known model organism in neuroscience that predominantly transmits information through non-spiking signals. These three neurons, arbitrarily labeled by convention as RIM, AIY and AFD, represent, to date, the three possible forms of non-spiking neuronal responses of *C. elegans*.

## Introduction

Spiking neurons are often considered as the major information processing unit of the nervous system. Nonetheless, not all neurons elicit spikes. While spiking neurons compress continuous inputs into digital signals for transmitting information via action potentials, non-spiking neurons modulate analog signals through graded potential responses. More specifically, the amplitude and waveform of the action potentials are essentially invariant with respect to the amplitude, duration, and waveform of the stimulus, unlike graded potentials which are stimulus-dependent (see S1 Fig) [1]. An advantage of the non-spiking response type is that it allows not to sacrifice information content [2]. A large variety of nervous tissues in both vertebrate and invertebrate species have revealed that a number of sensory, inter- and motorneurons function without eliciting spikes. Some examples are the human retina neurons [3], numerous interneurons in insects and crustaceans [4], the motorneurons of the Ascaris worm [5, 6], or most of the *C. elegans* neurons [7]. Non-spiking neurons have been found in sensorimotor and central pattern generator circuits, and proven to be central in neuronal integration [4] and to provide a determining mechanism for the control of motor behavior [8–10].

Despite their differences, non-spiking neurons use similar mechanisms to those of spiking neurons to transmit neuronal information: they both rely on the active and passive propagation of electrical signals. The cell membrane is also composed of similar ion channels, *i.e*. a large diversity of classical voltage-dependent ion channels have been experimentally and genetically identified in non-spiking neurons of different cell types [4]. As a consequence, several studies have already proposed conductance-based models (CBMs) as a means to characterize the non-spiking behavior of some neurons, such as retina neurons [11, 12] or *C. elegans* neurons [13]. These works are however built in an *ad-hoc* manner by combining both experimental measurements and results from different species and neurons. Unfortunately, such a procedure can easily fail to yield reliable conductance-based models [14, 15]. To the extent of our knowledge, this paper is a first attempt to propose general and systematic methods to characterize this type of neurons’ behavior using CBMs.

CBMs have become one of the most powerful computational approaches for characterizing the behavior of neurons [16]. In simple terms, a CBM is a biophysical representation of a neuron in which the ion channels are represented by conductances and the polar membrane by a capacitor [17, 18]. In such models, every individual parameter and state variable has an established electrophysiological meaning so that their role in the neuron dynamics can be unequivocally identified. However, due to the difficulty to perform some experimental recordings (*e.g*. ionic conductances [19]), many modeling studies suffer from the lack of sufficient physiological data to determine all the parameter values. As a consequence, parameters are often tuned in an *ad-hoc* manner. Furthermore, when new biological recordings come into play, these models can typically suffer from good generalization capabilities (*i.e*. the ability to predict acceptable responses to stimuli not used while building the model) [20, 21]. In order to overcome these issues, we propose a new approach in which all the model parameters are simultaneously determined, from *macroscopic* data, by trading off the accuracy and the capability of generalization of the model.

**Fig 1:**
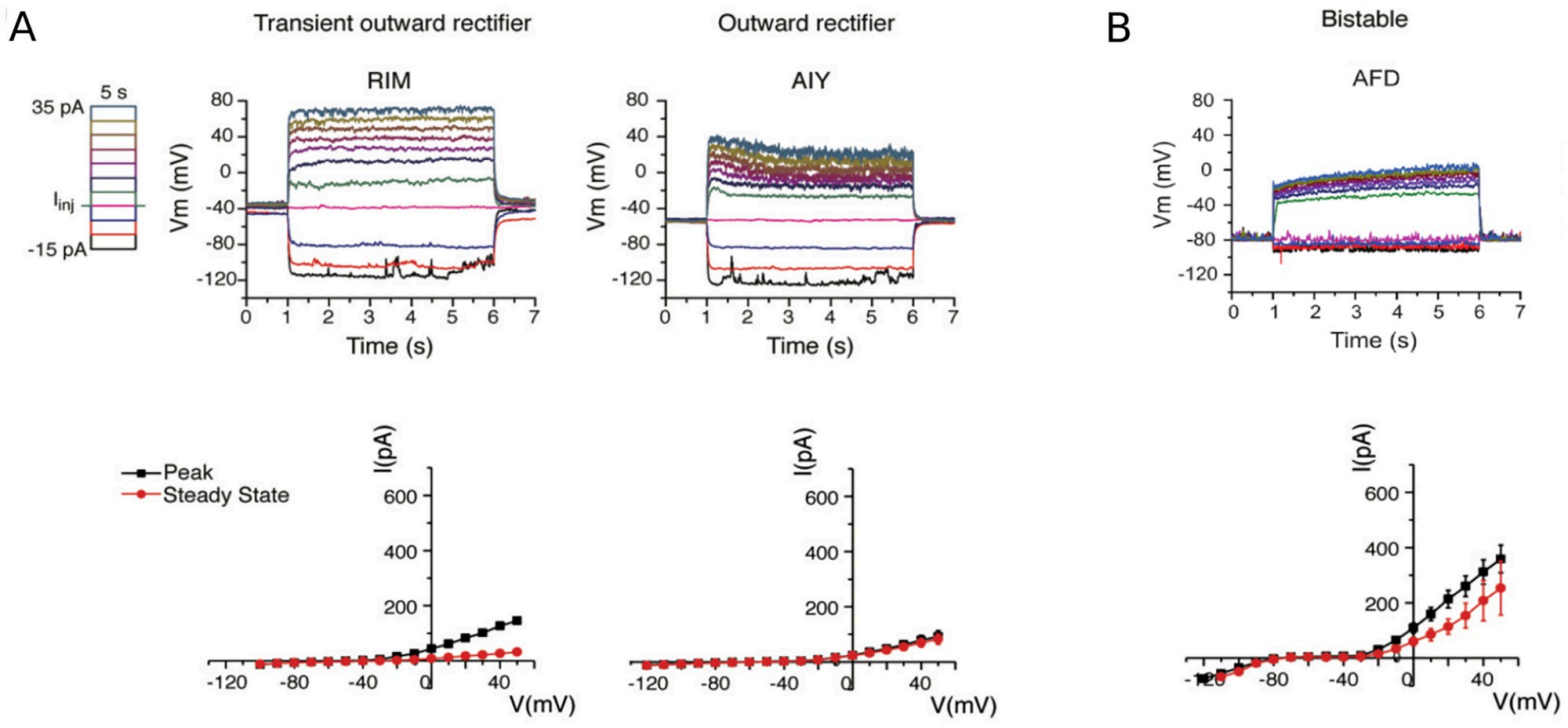
In-vivo recordings of three different non-spiking neurons of *C. elegans* which represent, to date, the three forms of possible non-spiking neuronal responses of the nematode. (Top) Evolution of membrane potential for a series of current injections, in spans of 5 seconds, starting from -15pA and increasing to 35pA by 5pA increments. (Bottom) I-V relationships obtained from averaged whole-cell current traces induced by a series of voltage steps in voltage-clamped RIM, AIY and AFD neurons (RIM: n = 3; AIY: n = 7; AFD: n = 3). Peak currents are measured by the absolute maximum amplitude of currents within the first 100 ms of each voltage step onset, while steady-state currents are measured by the averaged currents of the last 50 ms of each voltage step. **(A)** Near-linear behavior. Published in [23] (reproduced with the consent of the authors). **(B)** Bistable behavior. New unpublished results for AFD.

To obtain a CBM that characterizes the neuron behavior accurately and with a good generalization capability, one needs to capture the right underlying bifurcation structure of the neuron, *i.e*. the qualitative changes that the neuron behavior undergoes as a result of a change in stimuli. In a sense, *neurons are dynamical systems* [22]. In this paper, we show that the steady-state current (depicted in Fig 1) plays a pivotal role in the dynamics of non-spiking CBMs by determining: (i) the number of equilibria as well as their values, and (ii) all the bifurcations of the resting state along with the values to which they occur. Therefore, this paper adopts a multi-objective optimization approach so that, in addition to fitting the membrane potential evolution, it also captures the underlying bifurcation structure of non-spiking neurons by considering an additional objective: the fitting of the steady-state current.

In the present work, we apply our proposed approach on three non-spiking neurons (RIM, AIY and AFD) of the nematode *C. elegans*. Non-spiking neurons can display two typical behaviors: (i) *near-linear*, with a smoothly depolarization or hyperpolarization from the resting potential, and (ii) *bistable*, with nonlinear transitions characterized by a voltage jump between the resting potential and a depolarized potential of higher voltage. In particular, RIM and AIY neurons display a near-linear behavior (Fig 1A) while AFD exhibits a bistable one (Fig 1B). In this way, our approach is applied on experimental behavior representative of the known types of non-spiking neurons.

## Materials and methods

This paper is primarily based on the experimental data obtained in [23] for the three neurons under study. Nonetheless, we also provide new unpublished experimental data on the evolution of the AFD membrane potential and a description of the conducted experimental protocol. In order to find accurate models from these data with generalization capabilities, we need to capture the right underlying bifurcation structure of neurons [22]. In this section, we describe the important role that the steady-state current plays when trying to capture the bifurcation dynamics of non-spiking neurons, and therefore the importance of considering it during the optimization process. That is why we introduce a novel multi-objective approach that takes into account both the evolution of the membrane potential and the steady-state current of the neurons.

### Electrophysiology

The used *C. elegans* strain was PY1322 oyIs18[gcy-8::GFP] X with GFP exclusively expressed in AFD neurons. Experiments were performed on young adult hermaphrodites (3–4 days old) maintained at room temperature (22–23°C) on nematode growth medium (NGM) plates seeded with E. coli OP50 bacteria as a food source [24]. Electrophysiological recording was performed as previously described [23]. Briefly, an adult was immobilized with cyanoacrylate adhesive (Vetbond tissue adhesive; 3M) on a Sylgard 184-coated (Dow Corning) glass coverslip and dissected to expose AFD. Recordings were performed using single-electrode whole-cell current clamp (Heka, EPC-10 USB) with two-stage capacitive compensation optimized at rest, and series resistance compensated to 50%. The standard pipette solution was (all concentrations in mM): [K-gluconate 115; KCl 15; KOH 10; MgCl2 5; CaCl2 0.1; Na2ATP 5; NaGTP 0.5; Na-cGMP 0.5; cAMP 0.5; BAPTA 1; Hepes 10; Sucrose 50], with pH adjusted with KOH to 7.2, osmolarity 320–330 mOsm. The standard extracellular solution was: [NaCl 140; NaOH 5; KCL 5; CaCl2 2; MgCl2 5; Sucrose 15; Hepes 15; Dextrose 25], with pH adjusted with NaOH to 7.3, osmolarity 330–340 mOsm. Liquid junction potentials were calculated and corrected before recording. Data analysis were conducted using Fitmaster (Heka) and exported to OriginPro 2018 (OriginLab) for graphing.

### Conductance-based model description

Conductance-based neuron models, based on the Hodgkin-Huxley formalism, were first introduced in a series of seminal works in the 1950s [25]. They describe the neuronal dynamics in terms of activation and inactivation of voltage-gated conductances. In particular, the dynamics of the membrane potential *V* is described by a general equation of the form

$$\begin{array}{c} C\frac{dV}{dt}=-\sum_{ion}I_{ion}+I \end{array}$$ (1)

where *C* is the membrane capacitance, ∑_*ion*_ *I*_*ion*_ is the total current flowing accross the cell membrane, and *I* is an applied current.

The dynamics of every *I*_*ion*_ are governed by gating particles (gates) sensitive to the changes in the membrane potential (voltage). These gates can be of two types: activation gate and inactivation gate, each of which can be in an open or a closed state. The probability of an activation or inactivation gate being in the open state is denoted respectively by the variables *m* and *h*. Thus, the current generated by a large population of identical ion channels is given by

$$I_{ion}=g_{ion}m_{ion}^{a}h_{ion}^{b}\left(V-E_{ion}\right)$$

where *g*_*ion*_ is the maximal conductance (namely the conductance of the channel when all the gates are open); *E*_*ion*_ is the reverse potential, that is, the potential at which the ion current reverses its direction (a.k.a. equilibrium potential); and *a* and *b* respectively refer to the number of activation and inactivation gates. Channels that do not have inactivation gates (*b* = 0) induce a persistent current (*i.e*. current that does not inactivate) noted by *I*_*ion*,*p*_, while channels that do inactivate (*b* = 1) induce a transient current (*i.e*. current that inactivates) noted by *I*_*ion*,*t*_.

The dynamics of variables *m* and *h* are described by the following equation:

$$\begin{array}{c} \frac{dx}{dt}=\frac{x_{\infty }\left(V\right)-x}{\tau_{x}},\;x\in \left\{m,h\right\}. \end{array}$$

where *τ*_*x*_ is the *constant* time for which *x* reaches its respective equilibrium value *x*_∞_. The latter is expressed by a Boltzmann sigmoid function:

$$x_{\infty }\left(V\right)=\frac{1}{1+\text{exp}(\frac{V_{1/2}^{x}-V}{k_{x}})},\;x\in \left\{m,h\right\}.$$

where $V_{1/2}^{x}$ satisfies $x_{\infty }\left(V_{1/2}^{x}\right)=1/2$ and *k*_*x*_ is the slope factor with *k*_*m*_ > 0 and *k*_*h*_ < 0 as to represent activation and inactivation respectively, *i.e*., smaller values of |*k*_*x*_| lead to a sharper *x*_∞_.

### Conductance-based models for the RIM, AIY and AFD *C. elegans* neurons

From vertebrate to invertebrate species, non-spiking neurons are ubiquitous in nervous systems [4]. Experimental and genetic evidence supports the existence of various types of ion channels in these types of neurons. For instance, a large number of ion channels have been identified in non-spiking retinal networks [26]. Regarding the *C. elegans* neurons, there is extensive biological evidence (refer to [16] and references therein) supporting the existence of calcium, inwardly rectifying potassium and potassium channels. In more detail, *C. elegans* genome sequencing [27], electrophysiological measurements [7], and calcium imaging [28, 29], combined with a series of in-silico experiments, identified the most suitable models for electrophysiology of RIM, AIY and AFD neurons [16] that we use in this paper as base models. Specifically, *I*_*Ca*,*p*_ + *I*_*Kir*_ + *I*_*K*,*t*_ + *I*_*L*_-model was identified for RIM and AFD neurons, and *I*_*Ca*,*t*_ + *I*_*Kir*_ + *I*_*K*,*p*_ + *I*_*L*_-model for AIY. A complete mathematical description of these models is presented in S1 Table.

### Bifurcation dynamics of non-spiking neurons

In typical voltage-clamp experiments, the membrane potential is stabilized at several values *V*_*H*_ (*H* stands for *hold*) for which the resulting currents are measured. Asymptotic values (*t* → ∞) of those currents, depending only on *V*_*H*_, are called steady-state currents and noted *I*_∞_(*V*_*H*_). Mathematically, the steady-state current *I*_∞_ is the total current ∑_*ion*_ *I*_*ion*_ flowing accross the cell membrane when gating variables *m* and *h* are at their equilibrium, *i.e*. *x* = *x*_∞_ where *x* ∈ {*m*, *h*}. Therefore, its analytical expression is defined as follows:

$$\begin{array}{c} I_{\infty }\left(V\right)=\sum_{ion}I_{ion\infty }\left(V\right) \end{array}$$ (2)

where

$$I_{ion\infty }\left(V\right)=g_{ion}m_{ion\infty }^{a}\left(V\right)h_{ion\infty }^{b}\left(V\right)\left(V-E_{ion}\right)$$

In non-spiking CBMs, we show that the curve *V* → *I*_∞_(*V*) defined in (2) plays a pivotal role in the system dynamics by determining: (i) the number of equilibria as well as their values, and (ii) all the bifurcations of the resting state along with the values of *I* to which they occur. Indeed, any stationary point of gating variables *x* ∈ {*m*, *h*} must satisfy x_*_ = *x*_∞_(V_*_). Replacing this into the first equation on *V*, fixed points V_*_ of such models are those that satisfy the equation

$$\begin{array}{c} I_{\infty }\left(V_{*}\right)=I. \end{array}$$ (3)

**Fig 2:**
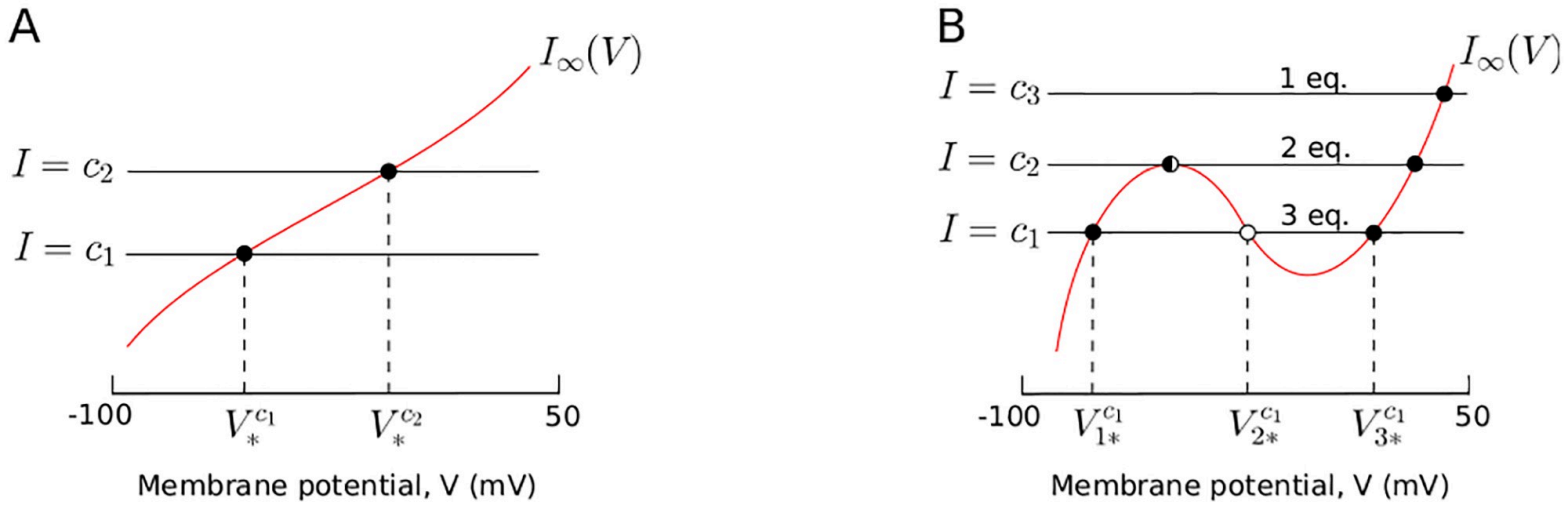
Two typical shapes of the steady-state current *V* → *I*_∞_(*V*), in red. Intersections of *I*_∞_ and horizontal line *I* = *c* (with *c* constant) correspond to equilibria of the system. We denote stable equilibria as filled circles ●, unstable equilibria as open circles ○ and saddle-node equilibria as ◐. **(A)** Monotonic steady-state current. $V_{*}^{c_{1}}$ and $V_{*}^{c_{2}}$ correspond to equilibria for a current injection *I* = *c*_1_ and *I* = *c*_2_ respectively. **(B)** N-shape steady-state current. The number of equilibria of the system depends on the value of *I*. For the sake of readibility, we highlight equilibria only for *I* = *c*_1_, noted $V_{1*}^{c_{1}}$, $V_{2*}^{c_{1}}$ and $V_{3*}^{c_{1}}$.

In other words, equilibria V_*_ correspond to the intersections between the steady-state curve *I*_∞_ and a horizontal line *I* = *c* where *c* is a constant. There are two standard shapes of the steady-state curve *I*_∞_, monotonic and cubic (Fig 2), each involving fundamentally different neuro-computational properties for non-spiking neurons:

- As shown in Fig 2A, CBMs with a monotonic steady-state current only have one equilibrium for any value of *I*. Non-spiking neurons with such a steady-state current display a near-linear behavior characterized by smoothly depolarization or hyperpolarization from the resting potential, such as the RIM and AIY neurons (Fig 1A and Table 1).
- As shown in Fig 2B, a N-shape curve leads to a saddle-node bifurcation. When *I* = *c*_1_, there are 3 equilibria, noted $V_{1*}^{c_{1}}$, $V_{2*}^{c_{1}}$ and $V_{3*}^{c_{1}}$. Increasing *I* results in coalescence of two equilibria (the stable $V_{1*}^{c_{1}}$ with the unstable $V_{2*}^{c_{1}}$). The value *I* = *c*_2_, at which the equilibria coalesce, is called the *bifurcation value*. For this value of *I*, there exist 2 equilibria. For *I* > *c*_2_, for example *I* = *c*_3_, the system has only one equilibrium. In summary, when the parameter *I* increases, a stable and an unstable equilibrium approach, coalesce, and then annihilate each other. Non-spiking neurons with a N-shape steady-state current display a bistable behavior characterized by a voltage jump between the resting potential and a depolarized potential of higher voltage, such as the AFD neuron (Fig 1B and Table 1).

**Table 1:** Numerical values of the steady-state current of the RIM, AIY and AFD neurons displayed in Fig 1.

**mV**	**-120**	**-110**	**-100**	**-90**	**-80**	**-70**	**-60**	**-50**	**-40**
**RIM**	/	/	-12.2	-9.13	-6.57	-4.91	-3.57	-2.13	-0.807
**AIY**	-13.1	-10.4	-7.92	-5.89	-4.11	-2.69	-1.02	0.0211	1.17
**AFD**	/	-68.6	-49.5	-18.2	-5.06	2.19	3.37	2.52	2.68
**mV**	**-30**	**-20**	**-10**	**0**	**10**	**20**	**30**	**40**	**50**
**RIM**	0.229	1.46	4.27	7.46	11.8	17.2	21.6	27.1	32.5
**AIY**	3.1	7.32	14.2	22.4	31.5	43.2	54.5	69.5	82.4
**AFD**	5.97	14.6	33.4	60.2	85	114	152	208	254

As a consequence, it can be stated that the steady-state current determines: (i) the bifurcation structure of non-spiking neurons when *I* is considered as the bifurcation parameter, and (ii) the equilibrium values of their graded responses to a particular stimuli.

### Objective functions

#### Primary objective: Membrane potential

The primary objective of the proposed conductance-based models is to reproduce the evolution of the membrane potential depicted in Fig 1 for the different neurons under study. To that end, we employ the cost function *f*_*V*_ as being the root-mean-square error normalized to the noise level (*i.e*. standard deviation) of each experimental voltage trace. The noise level, noted *σ*_*I*_, is estimated as in [30], that is, we choose a time window at the end of each trace where the curve is relatively flat for calculating the standard deviation. Therefore, *f*_*V*_ takes the following form:

$$\begin{array}{c} f_{V}\left(\theta_{V}\right)=\frac{1}{\left|I\right|}\sum_{I}\frac{\sqrt{\frac{1}{N}\sum_{t}{\left(V_{exp}\left(I,t\right)-V_{\theta_{V}}\left(I,t\right)\right)}^{2}}}{\sigma_{I}} \end{array}$$ (4)

where *V*_*exp*_(*I*, *t*) are the *experimental* voltages depicted in Fig 1 and $V_{\theta_{V}}\left(I,t\right)$ the voltages *estimated* by the model where *θ*_*V*_ is the vector containing all the model parameters (see S1 Table); *t* ∈ [0, 50*ds*] corresponds to the biological real time with a sampling period of Δ*t* = 0.004*ds*; *N* = 12500 is the number of data points in the measurement record, and *I* corresponds to successive step values of current injections starting from -15pA and increasing to 35pA by intervals of 5pA.

#### Secondary objective: Steady-state current

As the primary objective alone may fail to predict generalized responses to novel stimuli, the secondary objective aims to fit the mean of the experimental responses of the steady-state current (RIM: n = 3; AIY: n = 7; AFD: n = 3) displayed in Fig 1. The fitting of the steady-state current is carried out by minimizing the root-mean-square error normalized to the standard deviation, noted *σ*. Therefore, the cost function denoted *f*_∞_ is defined as follows:

$$\begin{array}{c} f_{\infty }\left(\theta_{SS}\right)=\frac{1}{|V_{H}|}\sum_{V_{H}}\frac{\sqrt{{\left(I_{\infty }^{exp}\left(V_{H}\right)-I_{\infty }^{\theta_{SS}}\left(V_{H}\right)\right)}^{2}}}{\sigma_{V_{H}}} \end{array}$$ (5)

where $I_{\infty }^{exp}\left(V\right)$ is the *experimental* mean (Fig 1) and $I_{\infty }^{\theta_{SS}}\left(V\right)$ the *estimated* one; *θ*_*SS*_ is the vector containing the parameters related to the steady-state current (see S1 Table); *V*_*H*_ corresponds to a series of voltage clamped starting from -100mV and increasing to 50mV by 10mV increments, and $\sigma_{V_{H}}$ the experimental noise level (standard deviation).

#### Initial conditions

About the initial conditions of the model, *V*_0_ is set to the biological values determined by Liu et al. [23]: *V*_0_ = −38mV for RIM, *V*_0_ = −53mV for AIY, and *V*_0_ = −78mV for AFD. Meanwhile, *m*_0_ and *h*_0_, because of the lack of biological information, are considered as two additional parameters to be estimated within the optimization procedure (*i.e*. along with ionic conductances and the other parameters). This is relevant for multistable systems, such as the bistable AFD neuron, which has two stable asymptotic states. For such systems, the convergence to a stable state depends on the initial conditions and a bad initialization choice could result in the inability of the system to fit data. Therefore, by considering *m*_0_ and *h*_0_ as parameters to be estimated within the optimization procedure, the choice of these initial conditions is robust as the unique equilibrium point of non-spiking CBMs is necessarily globally asymptotically stable [31].

#### Conflicting cost functions

Both primary and secondary objectives can be considered conflicting. In principle, there is no biological reason behind such a conflict, however, the different nature of the data employed in each of the functions, which are obtained from different experimental procedures with their own intrinsic and extrinsic sources of experimental noise, prevent finding a single optimal parameterization that optimizes both objectives at once. Therefore, the multi-objective approach proposed in this paper provides a natural mechanism for both objectives to be treated simultaneously.

### Differential evolution

Originally proposed by Storn and Price [32], differential evolution (DE) is a simple yet powerful evolutionary algorithm for global optimization, successfully applied in many practical cases [33]. In the context of parameter estimation in conductance-based models (as it is the case in this paper), it has not only been shown to be an effective method [16, 34], but also superior to other optimization methods such as *genetic algorithms*, *simulated annealing* and *particle swarm optimization algorithm* in terms of convergence speed, simulation time, and minimization of the cost function [35].

As every population-based metaheuristic, DE is an optimization method that iteratively optimizes a problem by trying to improve a set of *NP* candidate solutions, so-called individuals, that are initially set at random within a given solution space of *D* parameters. At each iteration, new individuals (called trial vectors) are constructed by means of two operations: so-called *mutation* and *crossover*. Then *selection* determines which individuals will survive into the next iteration. Every individual of the population has to serve once as target vector, so that there are *NP* competitions in one generation and the population size is kept constant at *NP* with *NP* ≥ 4. During the mutation operation, if a component of a mutant vector falls out of the bounds of the feasible region (depicted in Table 2), we set this component to the closest boundary value. This approach is particularly efficient if the optimum lies near bounds and produces feasible solutions by making as few alterations to the mutant vector as possible; unlike other techniques consisting in random reinitialization or penalty [36].

**Table 2:** Parameter bounds, determined to be biologically relevant [16, 22, 23].

Parameters	Minimum value	Maximum Value
*g*_*Ca*_, *g*_*Kir*_, *g*_*K*_, *g*_*L*_	0*nS*	50*nS*
*E*_*Ca*_	20mV	150mV
*E*_*K*_	-100mV	0mV
*E*_*L*_	-80mV	30mV
$V_{1/2}^{m},V_{1/2}^{h},V_{1/2}^{Kir}$	-90mV	0mV
*k*_*m*_	0mV	30mV
*k*_*h*_, *k*_*Kir*_	-30mV	0mV
*τ*_*m*_, *τ*_*h*_	0ds	15ds
$x_{m}^{0}$ , $x_{h}^{0}$	0	1
*C*	0	10

### Multi-objective proposal

In this paper, the conflicting nature of the proposed primary and secondary objectives imposes a multi-objective treatment of the problem since, under two or more conflicting objectives, there is not a single optimal solution that can optimize all objectives simultaneously. Instead, in a multi-objective setting, solutions can be compared by using the notion of *dominance*: a solution *A* is said to be dominant over another solution *B* if *A* is superior to *B* in at least one objective while *B* is not superior to *A* in the rest of objective functions. Using this notion, the multi-objective outcome is not one but a set of non-dominated optimal solutions, so-called the Pareto front.

Out of all variants of DE for solving multi-objective optimization problems [37], the DEMO (Differential Evolution for Multi-objective Optimization) approach [38] is selected because it provides a good trade-off between the simplicity of the implementation and the good results on benchmarks compared to several state-of-the-art methods in terms of convergence and quality of the obtained solutions [37, 38].

Using DEMO as baseline algorithm, the proposed multi-objective approach has been tailored to best suit the nature of the problem, where the primary objective (membrane potential) must prevail over the secondary one (steady-state current). In other words, the primary objective must be favored as it is the one that guarantees quality in the neuronal response while the secondary objective is aimed at capturing the bifurcation structure of the neuron model as to improve its generalization capabilities. We denote the proposed approach DEMO/rand/best/biased.

Inspired by multi-objective guided search [39], the DEMO/rand/best/biased variant tries to guide the search towards an optimal region on the primary objective. To that end, in a preliminary step, a standalone single-objective DE is executed to yield a good candidate solution on the primary objective. This solution is then used to bias the multi-objective approach by integrating it into the initial randomly generated population. In order to reinforce this bias, the algorithm incorporates a rand/best strategy [40] that greedily uses the best individual on the primary objective to form the trial vector. The aim of this variant is therefore to concentrate and explore the Pareto front region around the best found primary objective solution. The consequence is that the algorithm provides a set of solutions that reproduce the evolution of the membrane potential with high fidelity due to the bias, while taking into account the bifurcation structure of the neuron guided by the secondary objective.

The DEMO/rand/best/biased algorithm was run with different values of control parameters *NP*, *F* and *CR* in order to fine-tune its search capabilities. The values that we recommend are *NP* = 600, *F* = 1.5 and *CR* = 0.3 with a number of 2000 iterations. The algorithm was run 10 times for each neuron model and combination of control parameters.

#### *Automated* decision-making process

The result of a multi-objective optimization process is a set of non-dominated solutions which constitute the best found trade-offs between the conflicting objective functions. If the aim is to adopt one of these solutions as a global solution to the problem, a decision-making process need to be put in place in order to discriminate the selected solution under some criteria. In order to automate this process, we propose a four-stage method that automatically selects a solution capable of reproducing adequate neuronal responses to new stimuli.

- **Step 1**: Split the membrane potential dataset into three sets.
  **Procedure**: The membrane potential dataset depicted in Fig 1 is split into three sets: the training set, the validation set, and the test set [41]. The training set, from which the model parameters are estimated, is composed of all the traces of membrane potential for the series of current injections going from -15pA to 25pA by 5pA increments and also the steady-state current. The validation set, used to select a solution with a good predictive capability, is composed of the voltage trace relative to 30pA. The test set, composed of the voltage trace relative to 35pA, is used to assess the model performance from data not used in any part of learning or decision-making process. The different sets are summarized in Table 3.
  The validation and test sets are selected from the voltage traces relative to the highest stimulus values for the following reasons:
  1. While simultaneously optimizing the steady-state current and the experimental voltage traces, the empirical evidence suggests that the conflicting nature of both objectives maximizes at the upper extreme values (refer to the Results section and Figures therein). In other words, the most aberrant behavior that can occur is for stimuli higher than those considered during the training procedure.
  2. We select high traces for validation or test, the deterioration of a voltage trace between two traces is necessarily constrained and cannot be as large as the one observed for stimuli higher than those used during the parameter estimation procedure.
- **Step 2**: Determining the set of non-dominated solutions.
  **Procedure**: 10 runs with different random seeds of the multi-objective optimization approach DEMO/rand/best/biased are conducted using the training set. The final set of solutions (that we denote as *S*) is composed of all non-dominated solutions found during these independent runs.
  **Input**: 6000 solutions (600 solutions per run × 10 independent runs).
  **Output**: A set *S* composed of all non-dominated solutions.
- **Step 3**: Selecting solutions with a correct bifurcation structure.
  **Procedure**: This step aims at eliminating from the set *S* the solutions that do not display the right expected shape of the steady-state current *I*_∞_, *i.e*. monotonic for the RIM and AIY neurons, and N-shape for AFD. To do so, we first compute the first-order derivative of *I*_∞_, noted $I_{\infty }^{\prime }$. For the RIM and AIY neurons, we then verify that $I_{\infty }^{\prime }\left(V\right)>0$ for any values of *V* ∈ [−100mV;50mV] to ensure the monotonicity of *I*_∞_. For the AFD neuron, $I_{\infty }^{\prime }$ has to be positive, then negative, and positive again to ensure the N-shape of *I*_∞_. These are the conditions we verify to select solutions with a correct bifurcation structure.
  **Input**: The set *S* composed of all non-dominated solutions.
  **Output**: A set *S*_1_ composed of all non-dominated solutions displaying appropriate bifurcation structure.
- **Step 4**: Selecting the best solution according to the validation trace.
  **Procedure**: Using Eq (4), compute the numerical scores of all solutions in *S*_1_ by only considering the validation trace. The solution with the lowest score, *i.e*. minimal cost function, is the one selected.
  **Input**: The set of non-dominated solutions *S*_1_ and the validation trace.
  **Output**: The final selected solution.

**Table 3:** Training, validation and test sets.

Training set	Validation set	Test set
• Voltage traces for stimuli going from −15pA to 25pA.	• Voltage trace relative to 30pA.	• Voltage trace relative to 35pA.
• Steady-state current.		

The proposed decision-making process does not take into account the test trace. The aim is to reserve a trace that has not been used in any part of the learning or decision-making process to assess the quality of the solution found.

## Results

A series of in-silico experiments is conducted with the purpose of showing the predictive capabilities of the proposed multi-objective approach (see Materials and methods). In addition to the fitting of the membrane potential, the proposal aims to capture the bifurcation dynamics of the neuron by considering the fitting of the steady-state current as a second objective. This section illustrates first the problems that a single-objective approach encounters when trying to generalize the responses of a neuron model to new stimuli. Then, the multi-objective approach is analyzed and shown to be capable of predicting adequate responses to the same new stimuli.

### Single-objective optimization may fail to determine a model with generalization capabilities

Single-objective optimization experiments are conducted using stimuli from -15pA and increasing to 25pA by 5pA increments, for the RIM, AIY and AFD neurons. The obtained parameter values for the three neurons are shown in S2 Table. The generalization capability is then assessed from the voltage trace relative to 30pA and 35pA.

#### The AFD case

**Fig 3:**
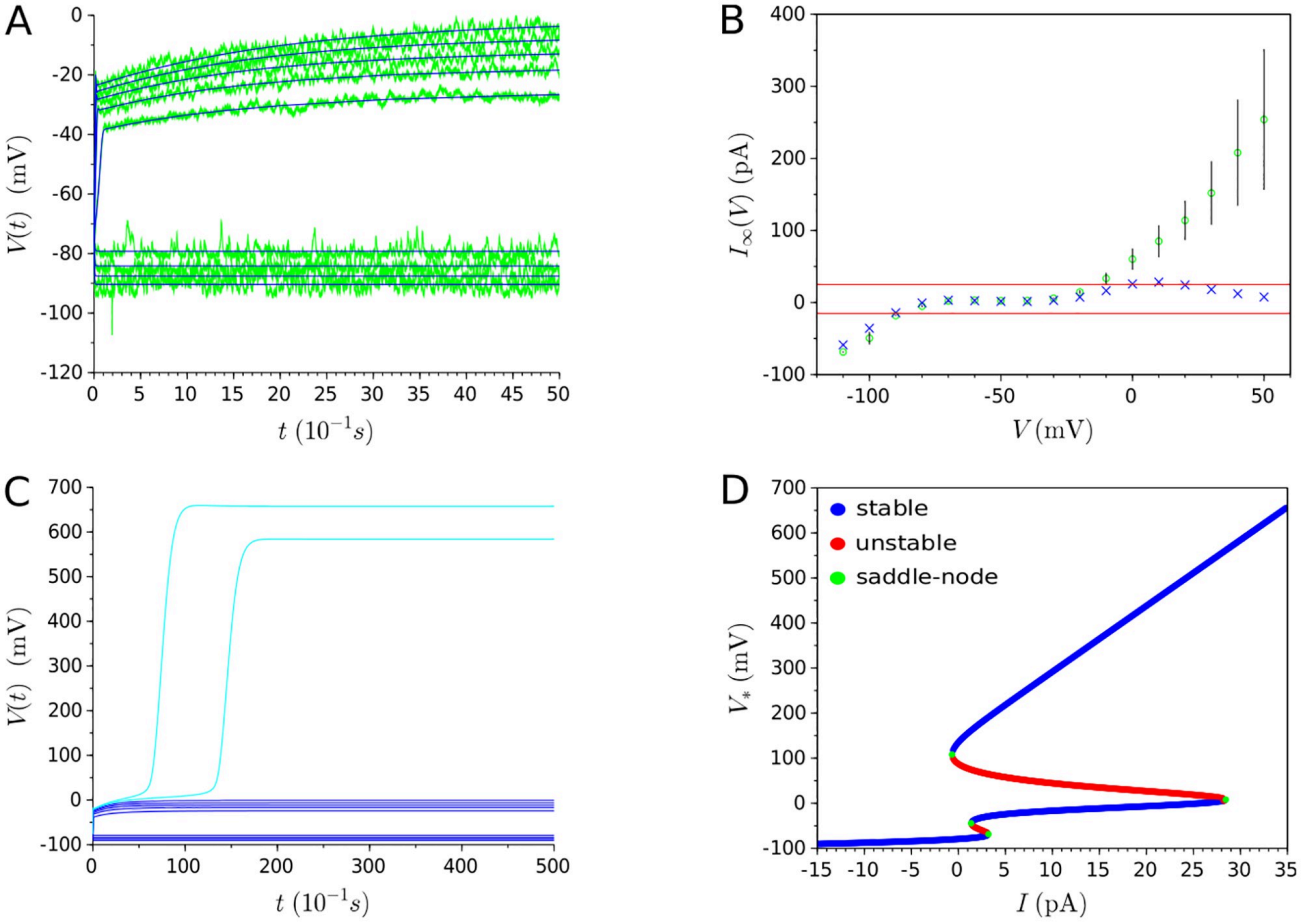
Results of single-objective optimization (evolution of AFD membrane potential). **(A)** Experimental data (represented in green) and *I*_*Ca*,*p*_ + *I*_*Kir*_ + *I*_*K*,*t*_ + *I*_*L*_-model (represented in blue) overlap for a series of current injection starting from -15pA and increasing to 25pA by 5pA increments. **(B)** Experimental steady-state currents (represented by green circles) and estimated steady-state currents (represented by blue crosses) resulting from the fitting of membrane potential evolution in (A). Red lines delineate the interval [-15pA; 25pA]. **(C)** Dark blue curves represent the evolution of membrane potential for the same values of current injection than in (A) (*i.e*. stimuli starting from -15pA and increasing to 25pA by 5pA increments), whereas light blue ones represent the drastic non-physiological change of voltage traces for novel stimuli (30pA and 35pA). Note the difference of scale regarding y-axis between (A) and (C). **(D)** Bifurcation diagram. Four saddle-node bifurcations occur at *I* ≈ −0.66pA, *I* ≈ 1.36pA, *I* ≈ 3.19pA, and *I* ≈ 28.4pA.

Fig 3 shows the results obtained for the AFD neuron using the single-objective approach. The high quality of the fitting, which takes into account current injections in the interval [−15*pA*;25*pA*], can be observed in Fig 3A. Nonetheless, when considering the resulting steady-state currents of the model in Fig 3B, it can be observed that the model deteriorates for values higher than 25pA, involving a non-physiological dramatic change in the neuronal dynamics. Fig 3C confirms this non-physiological response in the evolution of the membrane potential for the 30pA and 35pA traces that are not taken into account during the parameter estimation phase. In fact, as the steady-state current displays a second aberrant and unexpected N-shape for *I* > 25, another saddle-node bifurcation occurs at *I* ≃ 28.4 (see Fig 3D), explaining the drastic rise of the membrane potential trajectory to a new stable state of higher voltage. Thus, it can be concluded that the model fails to predict neuron responses to stimuli not encountered during the parameter estimation process, making it not acceptable and inadequate for the description of the AFD neuron behavior.

#### The near-linear RIM neuron

**Fig 4:**
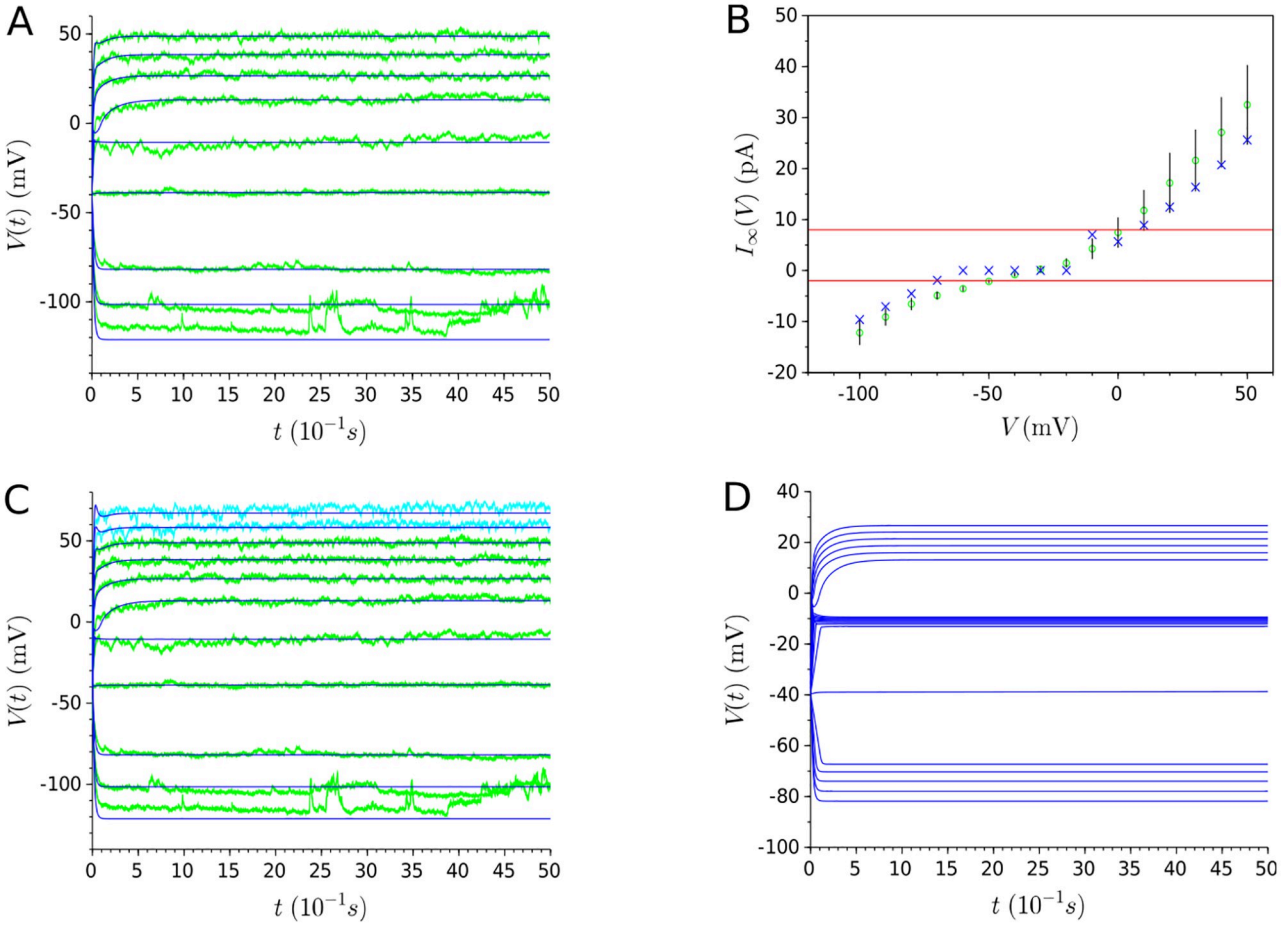
Results of single-objective optimization (evolution of RIM membrane potential). **(A)** Experimental data (represented in green) and *I*_*Ca*,*p*_ + *I*_*Kir*_ + *I*_*K*,*t*_ + *I*_*L*_-model (represented in blue) overlap for a series of current injection starting from -15pA and increasing to 25pA by 5pA increments. **(B)** Experimental steady-state currents (represented by green circles) and estimated steady-state currents (represented by blue crosses) resulting from the fitting of membrane potential evolution in (A). Red lines delineate the interval [-2pA;8pA] in which the steady-state current deteriorates. **(C)** Dark blue curves represent the model traces relative to stimuli from -15pA to 25pA by 5pA increments, whereas light blue experimental traces represent experimental traces relative to 30pA and 35pA. **(D)** Evolution of membrane potential for a series of current injection starting from -5pA and increasing to 15pA by 1pA increments. Numerous voltage jumps occur due to the two N-shape of the steady-current displayed in (B) between the red lines.

As in the case of AFD, Fig 4A illustrates that the model fits well with experimental data for all series of current injections considered during the optimization process (*i.e*. traces relative to stimuli from -15pA to 25pA by 5pA increments). Additionally, Fig 4B reveals that the steady-state current does not heavily deteriorate for stimuli higher than 25pA, so that the model should obtain relative good predictive capabilities for new stimuli. This fact is confirmed by Fig 4C which shows a good fitting for the validation traces (depicted in light blue). Nonetheless, if we analyze the steady-state current in the interval *I* ∈ [−2pA;8pA] (*i.e*. space between the two red lines in Fig 4B), we can observe a deterioration of the steady-state current shape: instead of a monotonic shape, two N-shape appear. As a consequence, two saddle-node bifurcations occur so that the membrane potential of the model does not display a near-linear behavior as expected, but various jumps arise (as illustrated in Fig 4D) making the model inadequate for the description of the RIM neuron behavior.

#### The near-linear AIY neuron

**Fig 5:**
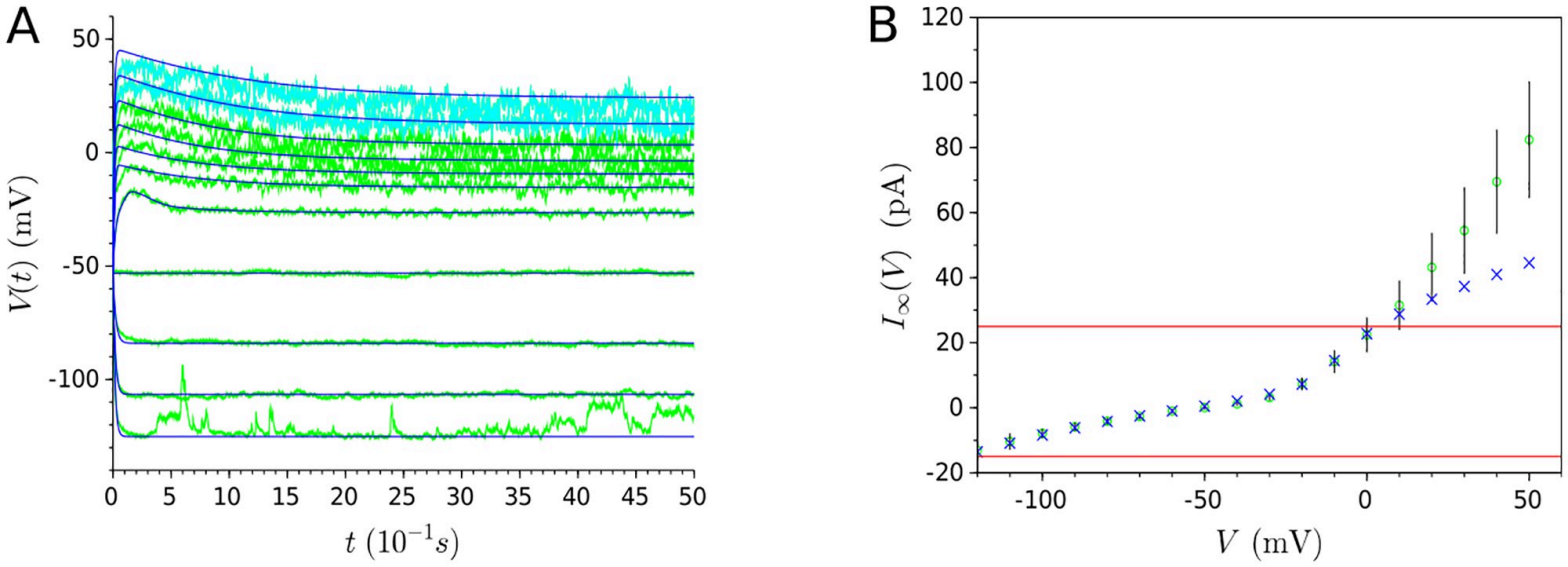
Results of single-objective optimization (evolution of AIY membrane potential). **(A)** Experimental voltages for stimuli starting from -15pA and increasing to 35pA by 5pA increments are represented in green. Estimated voltages resulting from the *I*_*Ca*,*t*_ + *I*_*Kir*_ + *I*_*K*,*p*_ + *I*_*L*_-model for stimuli going from −15pA to 25pA are represented in dark blue, whereas those relative to 30pA and 35pA are represented in light blue. **(B)** Experimental steady-state currents (represented by green circles) and estimated steady-state currents (represented by blue crosses) resulting from the fitting of membrane potential evolution in (A). Red lines delineate the interval [-15pA;25pA].

As can be seen in Fig 5A, the model is capable of predicting accurate responses for traces relative to 30pA and 35pA. However, one can observe a relatively high deterioration of the steady-state current for stimuli higher than 35pA (Fig 5B). One can then hypothesize that the model may not describe adequately the voltage responses for these stimuli.

### Obtaining non-spiking conductance-based models with generalization capabilities

In order to obtain a model with generalization capabilities, we follow the approach developed in the previous section. The DEMO/rand/best/biased algorithm is run with different values of control parameters *NP*, *F* and *CR* in order to fine-tune its search capabilities. The values that we recommend are *NP* = 600, *F* = 1.5 and *CR* = 0.3 with a number of 2000 iterations. For the three neurons, the model parameters obtained from the automated decision-making process described in the previous section are displayed in S2 Table.

#### Generalization capability of models

**Fig 6:**
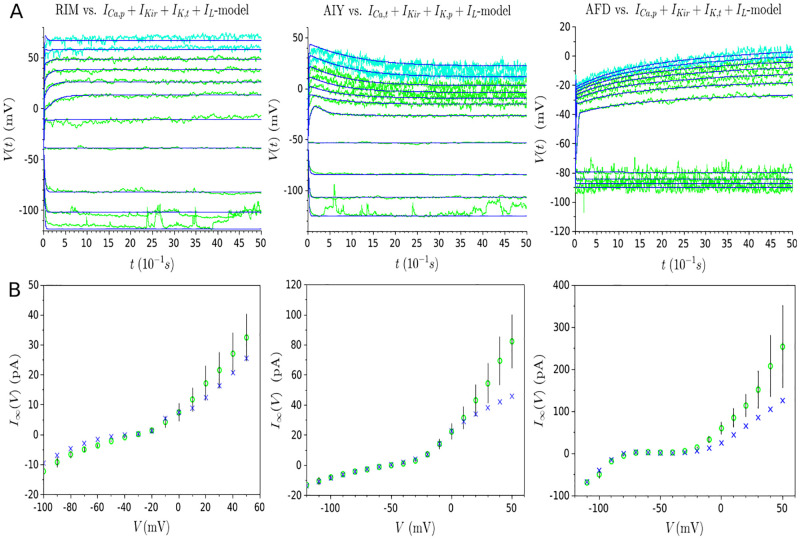
Results of multi-objective optimization for the RIM, AIY and AFD neurons. **(A)** Green traces represent the experimental membrane potential evolution for a series of current injections, in spans of 5 seconds, starting from -15pA and increasing to 25pA by 5pA increments. The light blue traces represent the validation and test set (*i.e*. traces relative to 30pA and 35pA). The dark blue traces represent the respective model for each neuron. **(B)** Experimental steady-state currents (represented by green circles) and estimated steady-state currents (represented by blue crosses) resulting from the multi-objectif optimization.

For each neuron under study, it can be observed in Fig 6A that the curves of the models fit well with experimental data in all series of current injections, including the test trace not used in any part of the model learning. The quality of the fitting is maintained throughout the entire evolution of the membrane potential. Furthermore, the steady-state current shape (Fig 6B), which determines the underlying bifurcation structure of non-spiking neurons, is captured for all neurons: a monotonic steady-state current for the RIM and AIY neurons, and a N-shape one for AFD. In this way, we constrain the RIM and AIY models to a near-linear behavior, and the AFD neuron to a bistable one, even in response to novel different stimuli not used during the model’s building. In the light of these results, it can be concluded that the proposed approach allows to get models with good generalization capabilities.

#### The steady-state current objective requires a relatively small deterioration to get models with predictive capabilities

**Fig 7:**
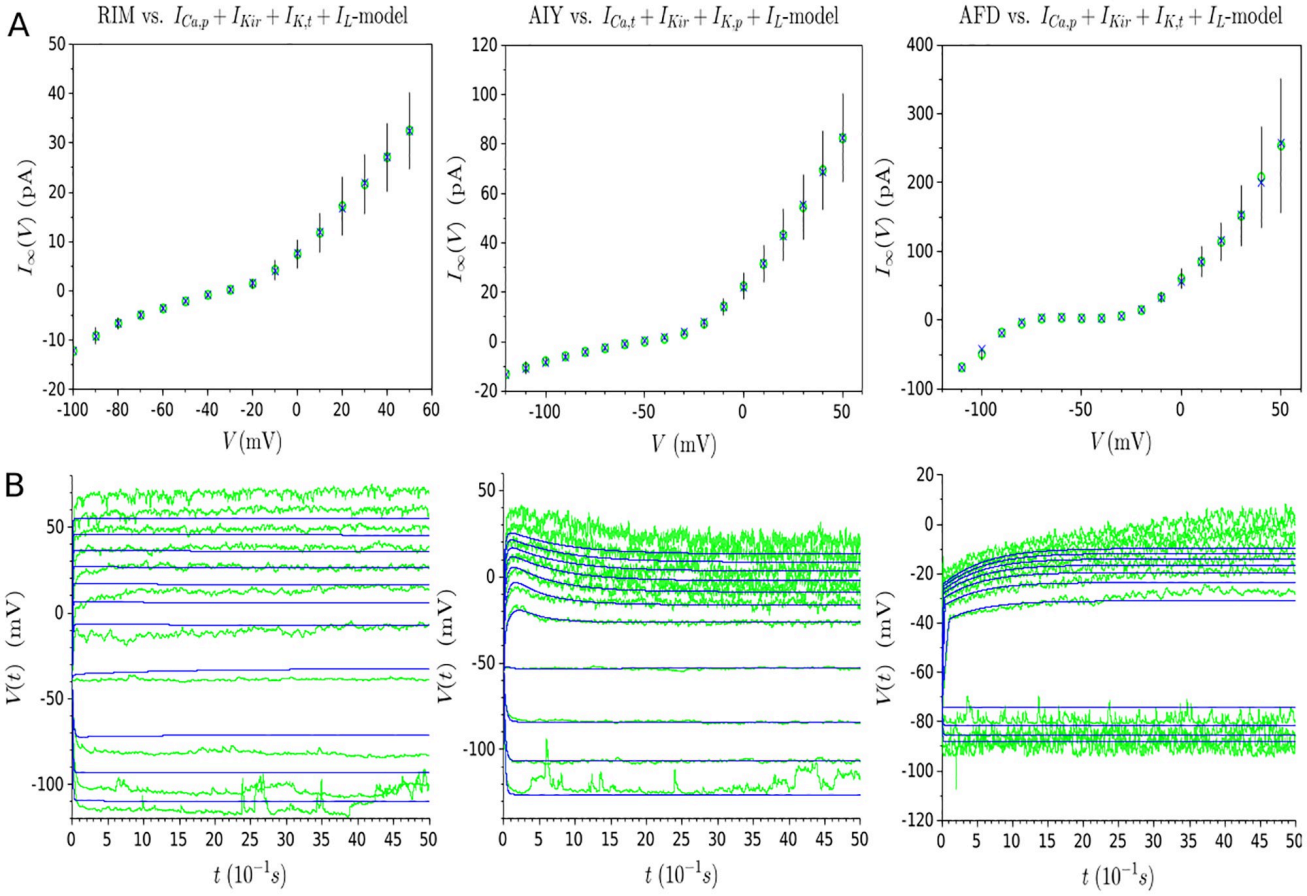
Solutions obtained from multi-objective optimization with perfect fitting of the steady-state current. **(A)** Experimental steady-state currents (represented by green circles) and estimated steady-state currents (represented by blue crosses). **(B)** Green (resp. blue) traces represent the experimental (resp. estimated) membrane potential evolution for a series of current injections, in spans of 5 seconds, starting from -15pA and increasing to 35pA by 5pA increments. Solutions with a perfect fitting of the steady-state current fail to describe the behavior voltage of neurons, showing that its deterioration is necessary to get adequate models.

Both objectives cannot be simultaneously optimized due to their conflicting nature. On the one hand, the steady-state curve for each neuron is obtained from the average of several different cells, while the membrane potentials are representative recordings from a single cell without averaging. On the other hand, the steady-state current and the voltage data are obtained from different experimental procedures with their own intrinsic and extrinsic sources of experimental noise [42–45]. Therefore, obtaining a perfect fitting of both objectives simultaneously is not feasible. Furthermore, the relative deterioration of the fitting for high steady-state currents in Fig 6B is correlated with higher values of the standard deviation at this level. Actually, these deteriorations are necessary to obtain models able to characterize voltage behavior. Indeed, as shown in Fig 7, a model that perfectly fits the steady-state current (Fig 7A) does not accurately reproduce the given voltage traces and fails to get the predictive capability (Fig 7B).

## Discussion

Some experimental techniques, such as ionic conductance measurements [19], are generally hard to perform. In the case of *C. elegans*, this difficulty is even higher due to the challenge of dissecting a one-millimeter long worm and then patching its small size neurons (1*μm* in the soma) [46]. The consequence is that obtaining detailed biological microscopic data from *C. elegans* neurons is a challenging task, and many neuronal parameters of *C. elegans* remain unknown to this day. In this paper, parameters are set to their biological values whenever they are known but, for the most part, they are simply bound to remain biologically plausible. Therefore, the optimization conducted in this paper aims to determine biologically plausible parameterizations that, formulated as hypotheses, would require future empirical validations. Despite the lack of such microscopic data, the macroscopic behavior of the model is equivalent to that of the neuron. Indeed, the methodology proposed in this paper, based on theoretical mathematical development and experimental validation, provides a systematic approach to endow the model with the same bifurcation structure as the neuron. As a consequence, and paraphrasing Eugene M. Izhikevich [22], “we can be sure that the behavior of the model is equivalent to that of the neuron, even if we omitted a current or guessed some of the parameters incorrectly”. In this section, we discuss both the biological and modeling implications of this methodology.

### On the modeling of non-spiking neurons in general

In this paper, our proposed approach was applied on various non-spiking *C. elegans* neurons, representative of the behavior of known types of non-spiking neurons (near-linear and bistable). Such neurons are not specific to *C. elegans* so that the proposed method could be straightforwardly applied to other non-spiking neuronal cell types. As stated in the introduction, this type of neurons are ubiquitous in a large variety of nervous tissues in both vertebrate and invertebrate species, *e.g*. in the human retina neurons [3], numerous interneurons in insects and crustaceans [4], the motorneurons of the Ascaris worm [5, 6], or most of the *C. elegans* neurons [7]. They have been found in sensorimotor and central pattern generator circuits, proven to be central in neuronal integration [4] and to provide a determining mechanism for the control of motor behavior [8–10].

### On the modeling of the *C. elegans*’ neuronal diversity

Numerous recordings of *C. elegans*’ neuronal activity have already been performed [7, 23, 47–55]. Liu *et al*. [23] classify the recorded neurons into four large distinct classes based on the features of the I-V curve (Fig 1). This classification is described in detail in Table 4. Among the different classes, the authors enumerate three types of non-spiking neurons, of which RIM, AIY and AFD are representative examples, and a fourth type involving the spiking neuron AWA. However, the electrophysiological properties of many *C. elegans* neurons are unknown yet, suggesting that additional types of neurons could be discovered in the future. The results presented in this paper show that the proposed method is capable of capturing the behavior of the current non-spiking neuronal diversity of *C. elegans*, and could be successfully applied to model new non-spiking neurons.

**Table 4:** Classification of the three types of non-spiking neurons in *C. elegans*, according to their current-voltage relationships. RIM, AIY and AFD neurons are representatives of class 1, 2 and 3 respectively.

Neuron classes	Class 1	Class 2	Class 3
Inward current features	Near-zero inward currents under hyperpolarizations.	Near-zero inward currents under hyperpolarizations.	Large sustained inwardly currents under hyperpolarizations.
Outward current features	Rapid inactivating outward currents under depolarizations: lack of large sustained currents.	Non-inactivating outward currents under depolarizations.	Large inactivating outward currents under depolarizations.
Neurons	**RIM** [23] AVA [50, 51, 54] PLM [47] AVE [51]	**AIY** [23, 48] VA5 [53, 54] VB6 [53, 54]	**AFD** [23, 49] ASER [7] RMD [50] AWC [49] ASH [52] AIA [55]

### On the modeling of the *C. elegans*’ nervous system

Due to its fully mapped connectome and its small number of neurons, the *C. elegans* nervous system serves to investigate how behavior emerges from its underlying physiological processes [56–58]. Modeling the nervous system of *C. elegans* involves two fundamental stages [59]: one relative to the modeling of the neuronal connectivity (connectome) and the other relative to the modeling of the neuronal dynamics. Nowadays, the vast majority of modeling works on *C. elegans* nervous system employ the well-established connectome but they do not take into account the specificities of the neuronal dynamics [59–69]. Typically, these works rather consider: (i) homogeneous model parameters for each neuron of the network (while *C. elegans* neurons display a large repertoire of behaviors), and (ii) a neuron model that do not correspond to the behavior of *C. elegans* neurons. The discordance between the accuracy of the connectome and the inaccuracy of the neuronal dynamics considered is explained by the lack of biophysical information for most neurons, making the building of conductance-based model adapted to *C. elegans*’ neuronal dynamics currently challenging [57]. As pointed out by Sarma *et al*. [57], building such neuron models is a key remaining component to make *C. elegans* nervous system modeling studies adequate for biological research.

In particular, we would like to emphasize an open problem where computational works could play an important role in order to fully understand the flow of information within the nematode’s nervous system [70]. If one wants to deepen further our understanding of the *C. elegans* nervous system, it is of paramount importance to gather information about the features of its synaptic connections, such as their intrinsic nature (excitatory or inhibitory) and their strength [70]. Actually, the connectome does not unveil such information [71]. To address that issue, some computational studies [60, 62, 64, 66–68] adopt an evolutionary approach in which the algorithm determines both the strength and nature of connections in order to obtain observable, realistic worm behavior. In such studies, the functional circuits studied are made up of identical neuron model parameters irrelevant to characterize the heterogeneity of *C. elegans* neurons and to represent acceptably their behavior (*e.g*. the homogeneous Izhikevich spiking model [72] is considered in [64, 66], or the Hindmarsh-Rose spiking model in [68]). Therefore, even if the macroscopic behavior of *C. elegans* is accurately reproduced, the results on the strength and nature of neuron connections may not be biologically adequate. We argue that the current paper provides a systematic approach and method to build conductance-based models capturing the dynamics of non-spiking *C. elegans*’ neurons, so that the second stage relative to the *C. elegans* neuronal dynamics modeling can be fulfilled.

### On the multicompartmental conductance-based modeling

It is worth noting that characterizing a neuron as “spiking” or “non-spking” is only relative to the site of recording. The fact that a neuron is spiking in one part of its anatomy does not exclude that it may have non-spiking activity in other parts. For example, even in spiking neurons, the integrative life of the cell is *predominantly* performed through graded electrical activity via the dendrites [4, 73]. The complex geometry of the dendritic tree, combined with its active and passive membrane properties, play a key role in the way neurons integrate synaptic inputs. Therefore, dendrites strongly influence both the timing and probability of neuronal output [74, 75]. In order to take into account the heterogeneity of the dendritic morphology as well as the different electrical characteristics between the regions (a.k.a. compartments) of the neuron, numerous modeling studies [76–78] use multicompartmental conductance-based models, which allow to develop more realistic and morphologically accurate models. More specifically, in such a multicompartmental description, the structure of a neuron is divided into separate compartments such as the dendritic tree, soma, axon, and axon terminal. Each of these compartments have their own membrane potential and gating variables that determine the membrane current within the compartment. The dynamics for the membrane potential of each compartment follow an equation of the form (1) as the one in this paper, and the compartments are coupled via conductances that depend on the relative sizes of dendritic and somatic compartments [17]. In this context, the parameters of the CBMs composing the different compartments of the neuron could be straightforwardly estimated following the methodology presented in this paper, provided that multicompartmental data are available.

## Supporting information

S1 Fig Feature differences between spiking and non-spiking neurons. (PDF)

S1 Table Full-fledged material description. Description of mathematical models for the RIM, AIY and AFD neurons, as well as their respective set of parameters *θ* for the optimization process.
(PDF)

S2 Table Estimated parameters for each of the models. Parameter values for each of the models resulting from the mono and the mutlti-objective optimization.
(PDF)
