# Supplementary material for: Systematic generation of biophysically detailed models with generalization capability for non-spiking neurons
Source: PLoS One. 2022 May 13;17(5):e0268380. doi: 10.1371/journal.pone.0268380 (PMC9106219; doi:10.1371/journal.pone.0268380)
Supplement: S1 Fig — (PDF) [file pone.0268380.s001.pdf]

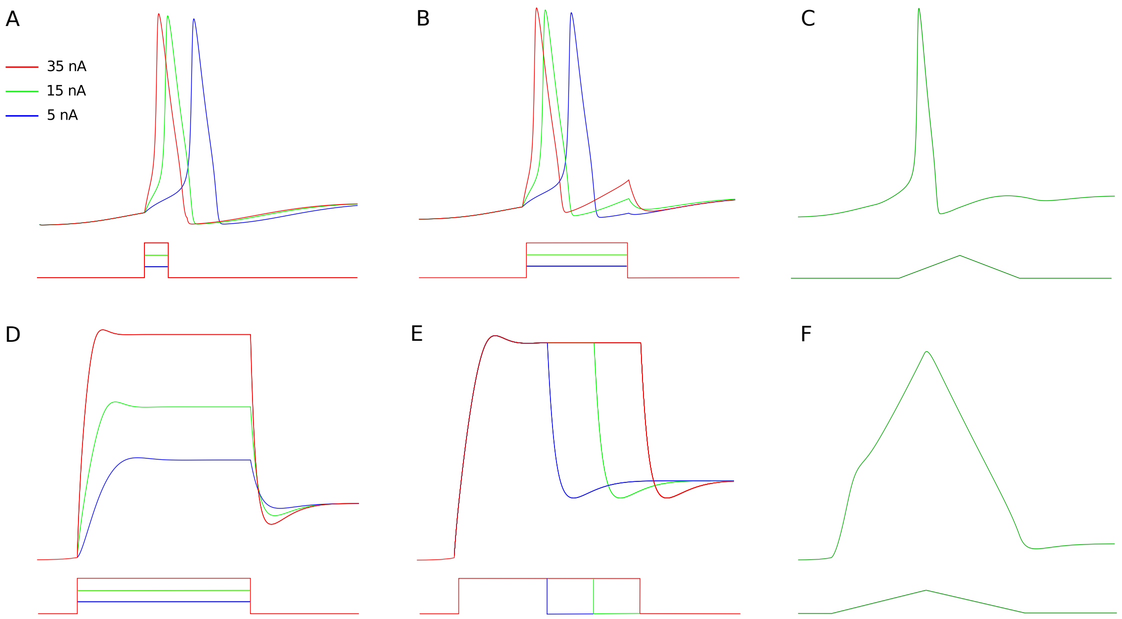

Figure 1: **Difference of features between action potentials (A-C) and graded potentials (D-F)**. Action potentials have been simulated from the classical Hodgkin-Huxley model, while graded potentials have been obtained by reducing its maximal conductances g_{Na} and g_K. The amplitude and waveform of the action potentials are essentially invariant with respect to the **(A)** amplitude, **(B)** duration, and **(C)** waveform of the stimulus, while the amplitude and waveform of the graded potentials are dependent on the **(D)** amplitude, **(E)** duration, and **(F)** waveform of the stimulus.
