# Supplementary material for: Systematic generation of biophysically detailed models with generalization capability for non-spiking neurons
Source: PLoS One. 2022 May 13;17(5):e0268380. doi: 10.1371/journal.pone.0268380 (PMC9106219; doi:10.1371/journal.pone.0268380)
Supplement: S1 Table — Description of mathematical models for the RIM, AIY and AFD neurons, as well as their respective set of parameters θ for the optimization process. (PDF) [file pone.0268380.s002.pdf]

$I_{Ca,p}+I_{Kir}+I_{K,t}+I_L$-model	$\begin{cases} C\dot{V} = -g_{Ca}m_{Ca}(V-E_{Ca}) - g_{Kir}h_{Kir\infty}(V)(V-E_K) - g_K m_K h_K(V-E_K) - g_L(V-E_L) + I \\ \dot{m_{Ca}} = \frac{m_{Ca\infty}(V)-m_{Ca}}{\tau_{m_{Ca}}},\ m_{Ca\infty}(V) = \left(1+\exp\left(\frac{V_{1/2}^{m_{Ca}}-V}{k_{m_{Ca}}}\right)\right)^{-1} \\ \dot{m_K} = \frac{m_{K\infty}(V)-m_K}{\tau_{m_K}},\ m_{K\infty}(V) = \left(1+\exp\left(\frac{V_{1/2}^{m_K}-V}{k_{m_K}}\right)\right)^{-1} \\ \dot{h_K} = \frac{h_{K\infty}(V)-h_K}{\tau_{h_K}},\ h_{K\infty}(V) = \left(1+\exp\left(\frac{V_{1/2}^{h_K}-V}{k_{h_K}}\right)\right)^{-1} \end{cases}$<br>$I_\infty(V_H) = g_{Ca}m_{Ca\infty}(V_H)(V_H-E_{Ca}) + g_{Kir}h_{Kir\infty}(V_H)(V_H-E_K) + g_K m_{K\infty}(V_H)h_{K\infty}(V_H)(V_H-E_K) + g_L(V_H-E_L)$<br>$\theta_V = [g_{Ca}\ g_{Kir}\ g_K\ g_L\ E_{Ca}\ E_K\ E_L\ V_{1/2}^{m_{Ca}}\ V_{1/2}^{Kir}\ V_{1/2}^{m_K}\ V_{1/2}^{h_K}\ k_{m_{Ca}}\ k_{Kir}\ k_{m_K}\ k_{h_K}\ \tau_{m_{Ca}}\ \tau_{m_K}\ \tau_{h_K}\ m_{Ca}^0\ m_K^0\ h_K^0\ C]$<br>$\theta_{SS} = [g_{Ca}\ g_{Kir}\ g_K\ g_L\ E_{Ca}\ E_K\ E_L\ V_{1/2}^{m_{Ca}}\ V_{1/2}^{Kir}\ V_{1/2}^{m_K}\ V_{1/2}^{h_K}\ k_{m_{Ca}}\ k_{Kir}\ k_{m_K}\ k_{h_K}]$
$I_{Ca,t}+I_{Kir}+I_{K,p}+I_L$-model	$\begin{cases} C\dot{V} = -g_{Ca}m_{Ca}h_{Ca}(V-E_{Ca}) - g_{Kir}h_{Kir\infty}(V)(V-E_K) - g_K m_K(V-E_K) - g_L(V-E_L) + I \\ \dot{m_{Ca}} = \frac{m_{Ca\infty}(V)-m_{Ca}}{\tau_{m_{Ca}}},\ m_{Ca\infty}(V) = \left(1+\exp\left(\frac{V_{1/2}^{m_{Ca}}-V}{k_{m_{Ca}}}\right)\right)^{-1} \\ \dot{h_{Ca}} = \frac{h_{Ca\infty}(V)-h_{Ca}}{\tau_{h_{Ca}}},\ h_{Ca\infty}(V) = \left(1+\exp\left(\frac{V_{1/2}^{h_{Ca}}-V}{k_{h_{Ca}}}\right)\right)^{-1} \\ \dot{m_K} = \frac{m_{K\infty}(V)-m_K}{\tau_{m_K}},\ m_{K\infty}(V) = \left(1+\exp\left(\frac{V_{1/2}^{m_K}-V}{k_{m_K}}\right)\right)^{-1} \end{cases}$<br>$I_\infty(V_H) = g_{Ca}m_{Ca\infty}(V_H)h_{Ca\infty}(V_H)(V_H-E_{Ca}) + g_{Kir}h_{Kir\infty}(V_H)(V_H-E_K) + g_K m_{K\infty}(V_H)(V_H-E_K) + g_L(V_H-E_L)$<br>$\theta_V = [g_{Ca}\ g_{Kir}\ g_K\ g_L\ E_{Ca}\ E_K\ E_L\ V_{1/2}^{m_{Ca}}\ V_{1/2}^{h_{Ca}}\ V_{1/2}^{Kir}\ V_{1/2}^{m_K}\ k_{m_{Ca}}\ k_{h_{Ca}}\ k_{Kir}\ k_{m_K}\ \tau_{m_{Ca}}\ \tau_{h_{Ca}}\ \tau_{m_K}\ m_{Ca}^0\ h_{Ca}^0\ m_K^0\ C]$<br>$\theta_{SS} = [g_{Ca}\ g_{Kir}\ g_K\ g_L\ E_{Ca}\ E_K\ E_L\ V_{1/2}^{m_{Ca}}\ V_{1/2}^{h_{Ca}}\ V_{1/2}^{Kir}\ V_{1/2}^{m_K}\ k_{m_{Ca}}\ k_{h_{Ca}} k_{Kir}\ k_{m_K}]$
