# Supplementary material for: Systematic generation of biophysically detailed models with generalization capability for non-spiking neurons
Source: PLoS One. 2022 May 13;17(5):e0268380. doi: 10.1371/journal.pone.0268380 (PMC9106219; doi:10.1371/journal.pone.0268380)
Supplement: S2 Table — Parameter values for each of the models resulting from the mono and the mutlti-objective optimization. (PDF) [file pone.0268380.s003.pdf]

	Single-objective			Multi-objective		
	**RIM**	**AIY**	**AFD**	**RIM**	**AIY**	**AFD**
g_{Ca}	0.68	0.124	0.06	0.27	0.136	6.7
g_{Kir}	0.254	0.157	2.02	0.1	0.156	2.32
g_K	1.16	0.223	6.05	0.02	0.22	28.01
g_L	0.0002	0.14	0.0001	0.22	0.14	0.004
E_{Ca}	20.16	135.9	146.05	42.9	127.4	23.61
E_K	-62.18	-98.23	-79.3	-98.01	-98.3	-79.26
E_L	-37.6	-41.07	-90	-55.75	-41.1	-89.43
$V_{1/2}^{m_{Ca}}$	-5.5	-19.09	-22.1	-2	-19.09	-3.65
$V_{1/2}^{h_{Ca}}$		-21.24			-21.28	
$V_{1/2}^{m_K}$	-9.38	-17.71	-2.83	-10.74	-17.99	-2.54
$V_{1/2}^{h_K}$	-65.7		-46.5	-3.49		-64.19
$V_{1/2}^{h_{Kir}}$	-24.27	-90	-84.16	-73.66	-89.95	-86.19
$k_{m_{Ca}}$	1.6	4.67	8.99	23.9	4.65	7.25
$k_{h_{Ca}}$		-17.62			-16.06	
k_{m_K}	1.28	7.39	9.99	2.27	7.41	8.44
k_{h_K}	-23.44		-24.21	-1		-29.36
$k_{h_{Kir}}$	-1.32	-30	-8.92	-1.43	-29.98	-9.01
$\tau_{m_{Ca}}$	0.399	0.0001	19.43	0.03	0.0001	5.62
$\tau_{h_{Ca}}$		10.59			11.12	
τ_{m_K}	0.03	0.0005	0.03	0.0006	0.001	0.0001
τ_{h_K}	0.61		6.16	3.3		0.045
m_{Ca}^0	0.002	0.001	0.002	0.16	0.33	0.001
h_{Ca}^0		0.80			0.78	
m_K^0	0.643	0.999	0.001	0.5	0.74	0.31
h_K^0	0.113		0.67	0.99		0.19
C	0.042	0.04	0.058	0.03	0.04	0.066
